# Development of a laboratorial platform for diagnosis of schistosomiasis mansoni by PCR-ELISA

**DOI:** 10.1186/s13104-018-3571-7

**Published:** 2018-07-11

**Authors:** Carolina Senra, Luciana Inácia Gomes, Liliane Maria Vidal Siqueira, Paulo Marcos Zech Coelho, Ana Rabello, Edward Oliveira

**Affiliations:** 1 Clinical Research and Public Politics in Infectious and Parasitic Diseases, Instituto René Rachou, Oswaldo Cruz Foundation (IRR/FIOCRUZ), Av. Augusto de Lima, 1715, Belo Horizonte, MG 30190-002 Brazil; 2 Biology of the Schistosoma mansoni and its Interaction with Host, Instituto René Rachou, Oswaldo Cruz Foundation (IRR/FIOCRUZ), Av. Augusto de Lima, 1715, Belo Horizonte, MG 30190-002 Brazil

**Keywords:** Schistosomiasis, *Schistosoma mansoni*, Diagnosis, PCR-ELISA, Disease control

## Abstract

**Objective:**

We developed a laboratorial platform to release a commercial platform used in the PCR-ELISA for the molecular diagnosis of schistosomiasis mansoni. On following, PCR-ELISA platform laboratorial was evaluated in 206 feces samples collected of individual living in a Brazilian low endemicity area.

**Results:**

The PCR-ELISA laboratorial platform indicated a prevalence rate of 25.2%, which was higher than the Kato-Katz technique (18.4%) and lower than the commercial platform (30.1%). Considering Kato-Katz technique as the reference, there were 97.4% and 91.1% of relative sensitivity and specificity rates, respectively. The laboratorial platform presented good precision, performance diagnostic, and can be used in replacement to the commercial platform for diagnosis of schistosomiasis by PCR-ELISA.

**Electronic supplementary material:**

The online version of this article (10.1186/s13104-018-3571-7) contains supplementary material, which is available to authorized users.

## Introduction

In Brazil, schistosomiasis is caused only by *Schistosoma mansoni* where was estimated 1% positive rate of schistosomiasis mansoni [1]. The laboratorial diagnosis is performed by the detection of *S. mansoni* using Kato-Katz technique [2] due to its practicability, efficacy, and low cost [3]. However, its sensitivity considerably decreases when used to diagnose cases post-treatment with praziquantel or in areas of low prevalence, where most individuals have low parasitic load [4, 5].

In this context, Pontes et al. [6] proposed a diagnostic by polymerase chain reaction (PCR) targeting a 110 base pair (bp) fragment from a 121 bp tandem repeat deoxyribonucleic acid (DNA) sequence from the *S. mansoni* genome [7]. Then, Gomes et al. [8] standardized a polymerase chain reaction-enzyme-linked immunoassay (PCR-ELISA) to detect the same DNA sequence using a commercial platform (PCR Plate Detection Kit; Sigma-Aldrich, St. Louis, MI, US). Besides being capable of processing a large number of samples, this assay had sensitivity of 97.4%, specificity of 85.1%, and provided an analytical sensitivity of 1.3 fg of DNA of *S. mansoni*, which is equivalent to 0.15 eggs/g of feces. However, the PCR Plate Detection Kit was discontinued by the manufacturer. In this study, we aimed to establish such test by developing a PCR-ELISA laboratorial platform for the molecular diagnosis of schistosomiasis mansoni.

## Main text

### Methods

This work was performed in two phases: (i) In a proof of concept phase, we standardized the PCR-ELISA laboratorial platform using genomic DNA of adult *S. mansoni* obtained from livers of Swiss albino mice 60 days after infection with 150 cercariae. The animals and cercariae were provided by the IRR/FIOCRUZ. (ii) In a phase II evaluation, the assay was validated in total DNA extracted stool samples from individuals living in the community of Pedra Preta, a rural locality of Montes Claros municipality considered a low endemicity area for schistosomiasis mansoni in the North of the state of Minas Gerais, Brazil. The population was constituted of 69 children (female/male: 33/36; age range of 1–17 years) and 137 adults (female/male: 66/71; age range of 18–86 years). We describe the step-by-step development of the PCR-ELISA platform laboratorial below.

### Proof of concept phase

Genomic DNA was extracted from adult *S. mansoni* using QlAamp DNA Mini and Blood Mini Handbook (QIAGEN, GmbH, Hilden, GE), according to the manufacturer’s protocol. The DNA concentration and purity were measured at 260 and 260/280 nm in a spectrophotometer Nanodrop ND-1000 (Thermo Fisher Scientific, Wilmington, DE, US). A highly repetitive genome sequence of parasites from the genus *Schistosoma* [7], (Gen Bank M61098) was amplified by PCR following the protocol described by Gomes et al. [8]. In each PCR assay, we included a negative control (PCR mix without DNA), and genomic DNA extracted from adult worms diluted 1:5 in linear acrylamide diluted in water (1:166 v/v) was used as a positive control.

The best PCR-ELISA laboratorial platform protocol was defined. The wells of the MaxiSorp^®^ polystyrene microplates (Nunc ThermoScientific, Vernon Hills, IL, US) were coated with 100 µl of streptavidin (Sigma-Aldrich, Co.), diluted to 5 μg/ml in 0.1 M phosphate buffer saline (PBS, pH 7.2), and incubated in a humid chamber at 37 °C for 1 h, followed by incubation in the refrigerator (2–8 °C) overnight. Next, the wells were washed four times with PBS containing 0.05% Tween (Sigma Chemical Co.) (PBS-T) and incubated for 2 h at 37 °C with 200 μl of 3% bovine serum albumin (BSA; Sigma Chemical Co.) solution in PBS-T. The wells were then washed four times with PBS-T, and 100 μl of PCR product diluted 1:25 in PBS were placed in duplicate into the wells of the microplates and incubated in a humid chamber at 37 °C for 30 min. The wells were washed three times with PBS-T, 100 μl of 0.1 M NaOH were added, and the plate incubated at room temperature for 10 min. The plate was washed once with 0.1 M NaOH and three times with 0.1 M Tris–HCl. Afterwards, 0.4 pmol fluorescein 5′-labeled probe (5′-TGGTTTCGGAGATACAACGA-3′; Integrated DNA Technologies Inc., Coralville, IO, US) diluted in 200 μl of hybridization solution [70% SSPE5X (0.75 M sodium chloride, 0.05 M sodium phosphate, 0.005 M EDTA), 30% Formamide and 0.1% SDS] was added to each well, followed by incubation in a humid chamber at 37 °C for 1 h. The wells were washed three times with 6× SCC solution (0.9 M NaCl and 0.09 M sodium citrate) and twice with 3× SCC solution (0.45 M NaCl and 0.045 M sodium citrate 0.1% SDS). Next, 150 μl of a solution containing 1% BSA in PBS were added and the microplates incubated for 30 min at 37 °C. Then, 150 μl of anti-fluorescein antibodies IgG conjugated to peroxidase (Invitrogen-Thermo Scientific, Wilmington, DE, US) diluted 1:5000 in a solution containing 1% BSA in PBS were added to each well of the microplate, which was incubated for 1 h in a humid chamber at 37 °C. Subsequently, the microplate was washed four times with PBS-T, and 100 µl of TMB (3,3′,5,5′-tetramethylbenzidine; Sigma Chemical Co.) were added, followed by incubation at room temperature for 5 min. The reaction was stopped by adding 50 μl of 1 N sulfuric acid solution per well. The absorbance reading was performed in a microplate reader (Model 550, Bio-Rad Laboratories, Hercules, CA, US) at the wavelength of 450 nm.

As an internal control, the human β-actin (*ACTB*) gene was amplified and detected using protocol described by Gomes et al. [8], and a negative control (PCR mix without DNA) was included. The cut-off of the PCR-ELISA laboratorial platform to define the presence or absence of the human *ACTB* gene was calculated using the mean absorbance reading of the negative PCR control (PCR mix without DNA) plus three standard deviations.

The lower limit of detection of PCR-ELISA laboratorial platform was defined by a positive control containing 3 ng of genomic DNA of adult worms successively diluted 1:10 in water containing linear acrylamide (994:6 v/v) to obtain 300 pg, 30 pg, 3 pg, 300 fg, 30 fg, and 3 fg of DNA. Considering that *S. mansoni* genome contain about 580 fg [9], these concentrations correspond to 517.2, 51.7, 5.17, 0.52, 0.052, 0.0052 times its genome. These samples were submitted to PCR according to Gomes et al. [8]. Then, the PCR products were analyzed on a 6% polyacrylamide gel and the PCR-ELISA laboratorial platform.

Precision tests (repeatability and reproducibility) were carried out with six DNA samples extracted from human feces (three negatives and three positives for the presence of *S. mansoni* eggs), according to the Kato-Katz technique. The repeatability test was performed by retesting four times the same samples in a single assay (intra-assay test). To measure the reproducibility, the same samples were tested in four separate trials in different days, by the same researcher (inter-assay test). The precision levels (repeatability and reproducibility) were measured by the variation coefficient (VC) of retesting the samples and using the equation: $\rm{VC}\, = \,\rm{Standard} \, \rm{deviation/Mean}\, \times \,100\%$

### Phase II evaluation

The assay was validated with 206 DNA samples previously extracted from stools that had been examined by Kato-Katz technique (12 slides) and PCR-ELISA platform commercial [7], employing the standardized conditions described above.

### Data analysis

The correlation between the absorbance readings and the Log10[*S. mansoni* DNA] was measured with Pearson’s coefficient and cut-off of the PCR-ELISA laboratorial platform was defined by a receiver operating characteristic curve (ROC Curve) analysis using GraphPad Prism 4.0 software (San Diego, CA, US). Taking the Kato-Katz technique (12 slides) as reference for schistosomiasis mansoni diagnosis, we compared the relative sensitivity and relative specificity rates obtained with the PCR-ELISA laboratorial and commercial platforms using a Chi square (Χ^2^) test in the OpenEpi, v. 3.01 [10], and considering a level of significance of *p *<0.05. Agreement among the Kato-Katz technique and laboratorial and commercial PCR-ELISA platforms was defined by Kappa index, and interpreted according to Landis and Koch [11].

## Results

**Fig. 1:**
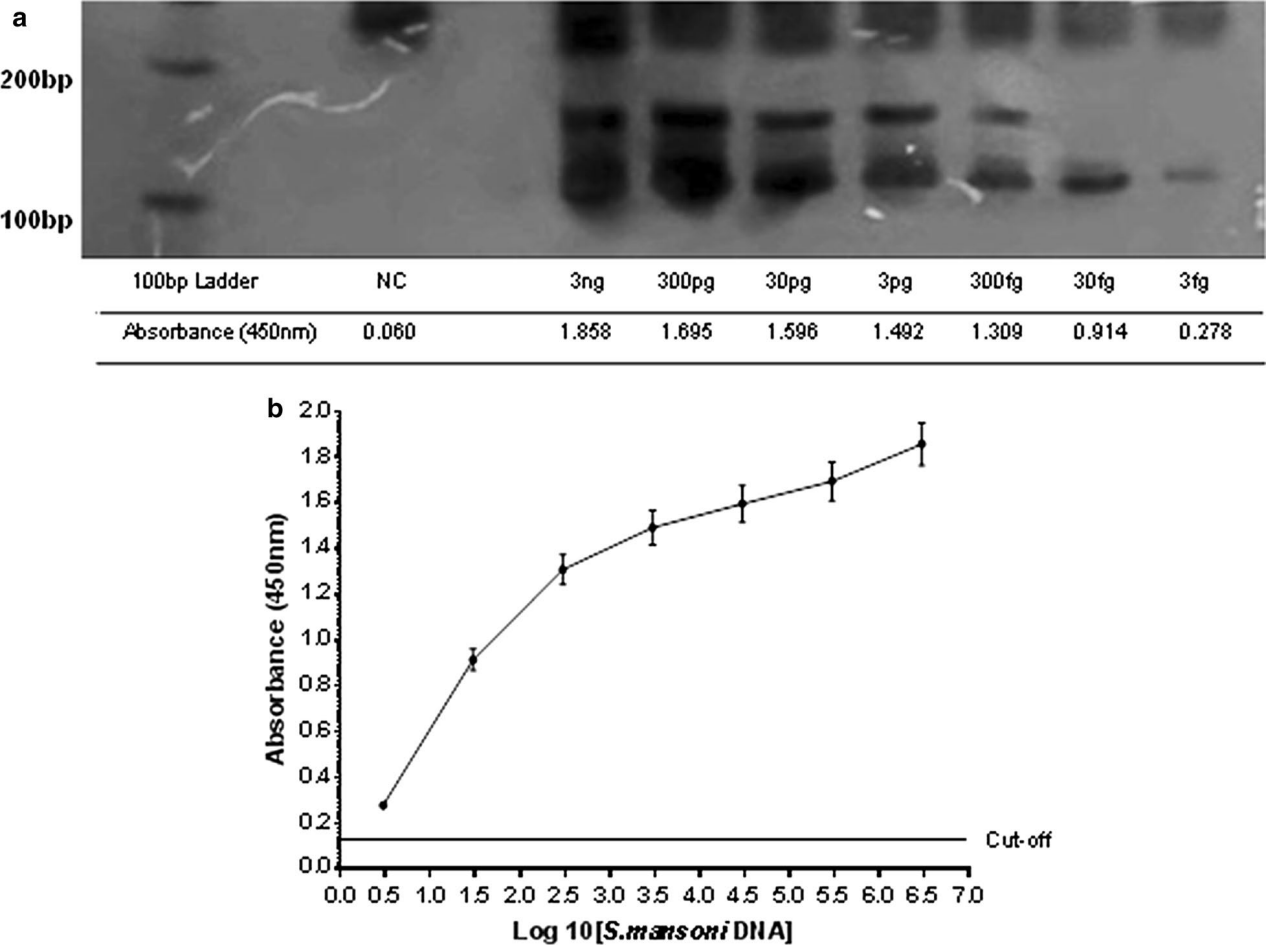
**a** 6% polyacrylamide gel showing 110 pb bands with decreasing intensity from 3 ng/µl to 3 fg/µl. The corresponding absorbance readings presented by the PCR-ELISA laboratorial platform are described below each lane, decreasing from 1.858 to 0.278, according to the concentration of *S. mansoni* DNA used in the PCR reaction. A 100 bp ladder marker (Promega, Madison, WI, USA) and a negative control (NC) are presented in the first two lanes. **b** Pearson’s positive correlation between the Log10 [*S. mansoni* DNA] and absorbance readings (450 nm). Pearson’s coefficient = 0.932 (r^2^ = 0.87*, p *= 0.002)

Our analytical sensitivity result regarding the PCR-ELISA laboratorial platform was of 3 fg of *S. mansoni* DNA, that equivalent to 0.0051 times the its genome [9]. Moreover, there was a positive correlation between the absorbance readings and the Log10[DNA *S. mansoni*], indicated by a Pearson’s coefficient of 0.932, (r^2^= 0.87*, p *= 0.002) (Fig. 1). Unspecific amplification was not a concern because the primers used in the PCR to diagnose schistosomiasis mansoni are genus-specific [8] and do not cross-react with DNA from *Ascaris lumbricoides*, *Ancylostoma duodenales*, *Taenia solium,* and *Trichiuris trichiuria* [11].

The VCs obtained by the intra-assay test for the negative control samples were 3.12, 1.94, and 7.68%, and the VCs for the positive control samples were 5.52, 2.72, and 0.82%. The VCs obtained by the inter-assay test for the negative control samples were 19.26, 15.37, and 13.26%, and for the positive control samples were 7.74, 6.28, and 5.86%.

Kato-Katz technique detected 38 individuals (38/206) with *S. mansoni* eggs in their feces, resulting in a prevalence rate of 18.4% in the studied area. Considering 0.136 as the cut-off point (area under the curve = 0.97), we established that the disease prevalence rate indicated by the PCR-ELISA laboratorial platform was 25.2% (52/206). This rate was higher than the 18.4% (38/206; *p *= 0.01) rate obtained with the Kato-Katz technique and was not significantly different from that obtained with the PCR-ELISA commercial platform (30.1%; 62/206; *p *= 0.13).

Only one sample (1/38) Kato-Katz positive presented negative result according to the laboratorial or commercial PCR-ELISA platforms, indicating a relative sensitivity of 97.4% (CI 95% 84.6–99.9). The negative samples in the laboratorial and commercial PCR-ELISA platforms were collected from two individuals, one who presented only two *S. mansoni* eggs per gram of faeces (EPG) and another who showed eight EPG by the Kato-Katz technique, respectively.

The relative specificity rates were 91.1% (CI 95% 85.4–94.7) and 85.1% (CI 95%: 79–89.7%) since 15 of the 168 samples diagnosed as negative by the Kato-Katz showed positive results by the laboratorial and commercial PCR-ELISA platforms. Ten samples presented positive results only by the PCR-ELISA commercial platform (Table 1). The agreement among Kato-Katz technique and PCR-ELISA laboratorial platform was considered good (K = 0.78). On other hand, the agreement of the laboratorial and commercial PCR-ELISA platforms results was considered excellent (K = 0.85) (Additional file 1: Table S1).

**Table 1:** Prevalence and relative sensitivity and specificity rates of the PCR-ELISA laboratorial and commercial platforms, taking Kato-Katz technique (12 slides) as reference for schistosomiasis mansoni diagnosis

Assays	Prevalence rate (CI 95%)	Relative sensitivity (CI 95%)	Relative specificity (CI 95%)
PCR-ELISA laboratorial platform (500 mg feces)	52/206 (25.2%) (19.5–31.8)	37/38 (97.4%) (86.5–99.5)	153/168 (91.1%) (85.8–94.5)
PCR-ELISA commercial platform (500 mg feces)	62/206 (30.1%) (23.9–36.9)	37/38 (97.4%) (86.5–99.5)	143/168 (85.1%) (79–89.7)

Difference between prevalence rates of the PCR-ELISA laboratorial and commercial platforms, *p *= 0.13

Difference between relative specificity rates of the PCR-ELISA laboratorial and commercial platforms, *p *= 0.03

## Discussion

In this study, we propose a PCR-ELISA laboratorial platform for diagnosis of schistosomiasis mansoni. The relative specificity rate and agreement of the PCR-ELISA laboratorial platform increased from 91.1 to 95.0% and 0.78–0.88 (data not shown) when 15 additional samples from individuals with discordant results were re-examined by Kato-Katz technique. These findings demonstrate the importance of evaluating a larger number of samples and a greater number of slides to reduce the number of false-negative results, since the limitation of the parasitological technique in detecting low parasitic burden is well established [4, 5].

Other encouraging aspects are that the PCR-ELISA laboratorial platform proposed herein showed performance similar to the molecular assays previously described [4, 12] with the advantage that it can be scaled to a large number of samples, and the results read objectively. Indeed, the PCR-ELISA laboratorial platform allows the processing of 46 samples per plate in duplicate and the results are read with a spectrophotometer. This processing requires considerably less time than the other conventional molecular or parasitological techniques to evaluate the same number of samples.

*Schistosoma* spp. has at least 600,000 copies of the 121 pb repetitive sequence in each cell, which constitutes 12% of the *S. mansoni* genome [13]. Therefore, in this work, all procedures were done by a trained researcher and physical barriers were utilized in all sample handling steps of the laboratorial and commercial PCR-ELISA platforms to minimize the risk of contamination. The possibility of PCR inhibition was discarded because all samples had absorbance readings ranging from 0.328 to 1.159 (mean = 0.810), which were higher than the cut-off point defined for detection of the *ACTB* gene (0.133). It is important to note that the present study report the internal validation results and still is necessary carry out multicenter studies in reference laboratories in order to externally validate the platform in these settings.

### Limitations

Ideally, parallel experiments should have been done to precisely compare the performance of the PCR-ELISA laboratorial and commercial platforms. However, as aforementioned, the commercial platform is no longer available for purchase.

## Additional file

**Additional file 1: Table S1.** Agreement among PCR-ELISA laboratorial platform, Kato-Katz technique and PCR-ELISA commercial platform.

## Abbreviations

PCR: polymerase chain reaction
ELISA: enzyme-linked immunosorbent assay
DNA: deoxyribonucleic acid
WHO: World Health Organization
