# Supplementary material for: Development of a laboratorial platform for diagnosis of schistosomiasis mansoni by PCR-ELISA
Source: BMC Res Notes. 2018 Jul 11;11:455. doi: 10.1186/s13104-018-3571-7 (PMC6042422; doi:10.1186/s13104-018-3571-7)
Supplement: Supplementary file 1 — Additional file 1: Table S1. Agreement among PCR-ELISA laboratorial platform, Kato-Katz technique and PCR-ELISA commercial platform. [file 13104_2018_3571_MOESM1_ESM.docx]

|  | | Kato-Katz technique  (500mg feces) | | |  | PCR-ELISA Commercial platform  (500mg feces) | | | |
| --- | --- | --- | --- | --- | --- | --- | --- | --- | --- |
| PCR-ELISA laboratorial  platform  (500mg  feces) |  | Positive | Negative | Total |  |  | Positive | Negative | Total |
|  | Positive | 37 | 15 | 52 |  | Positive | 51 | 1 | 52 |
|  | Negative | 1 | 153 | 154 |  | Negative | 11 | 143 | 154 |
| Total | | 38 | 168 | 206 |  | Total | 62 | 144 | 206 |
| Kappa index (K)  Confidence interval (CI) | | 0,78  (CI 95%: 0,63–0,91) | |  |  |  | 0,85  (CI 95%: 0,71–0,99) | |  |

Table S1: Agreement among PCR-ELISA laboratorial platform, Kato-Katz technique and PCR-ELISA commercial platform
